# A radiotherapy community data‐driven approach to determine which complexity metrics best predict the impact of atypical TPS beam modeling on clinical dose calculation accuracy

**DOI:** 10.1002/acm2.14318

**Published:** 2024-03-01

**Authors:** Fre'Etta Mae Dayo Brooks, Mallory Carson Glenn, Victor Hernandez, Jordi Saez, Hunter Mehrens, Julianne Marie Pollard‐Larkin, Rebecca Maureen Howell, Christine Burns Peterson, Christopher Lee Nelson, Catharine Helen Clark, Stephen Frasier Kry

**Affiliations:** ^1^ University of Texas MD Anderson UTHealth Graduate School of Biomedical Sciences Houston Texas USA; ^2^ Department of Radiation Physics University of Texas MD Anderson Cancer Center Houston Texas USA; ^3^ Department of Medical Physics Hospital Sant Joan de Reus, IISPV Tarragona Spain; ^4^ Department of Radiation Oncology Hospital Clinic de Barcelona Barcelona Spain; ^5^ Department of Biostatistics The University of Texas MD Anderson Cancer Center Houston Texas USA; ^6^ Department of Radiotherapy Physics University College London Hospital London London UK; ^7^ Department of Medical Physics and Bioengineering University College London London UK; ^8^ Medical Physics Department National Physical Laboratory Teddington UK

**Keywords:** beam modeling, complexity metrics, dose calculation accuracy, IMRT, MLC, quality assurance, VMAT

## Abstract

**Purpose:**

To quantify the impact of treatment planning system beam model parameters, based on the actual spread in radiotherapy community data, on clinical treatment plans and determine which complexity metrics best describe the impact beam modeling errors have on dose accuracy.

**Methods:**

Ten beam modeling parameters for a Varian accelerator were modified in RayStation to match radiotherapy community data at the 2.5, 25, 50, 75, and 97.5 percentile levels. These modifications were evaluated on 25 patient cases, including prostate, non‐small cell lung, H&N, brain, and mesothelioma, generating 1,000 plan perturbations. Differences in the mean planned dose to clinical target volumes (CTV) and organs at risk (OAR) were evaluated with respect to the planned dose using the reference (50th‐percentile) parameter values. Correlation between CTV dose differences, and 18 different complexity metrics were evaluated using linear regression; *R*‐squared values were used to determine the best metric.

**Results:**

Perturbations to MLC offset and transmission parameters demonstrated the greatest changes in dose: up to 5.7% in CTVs and 16.7% for OARs. More complex clinical plans showed greater dose perturbation with atypical beam model parameters. The mean MLC Gap and Tongue & Groove index (TGi) complexity metrics best described the impact of TPS beam modeling variations on clinical dose delivery across all anatomical sites; similar, though not identical, trends between complexity and dose perturbation were observed among all sites.

**Conclusion:**

Extreme values for MLC offset and MLC transmission beam modeling parameters were found to most substantially impact the dose distribution of clinical plans and careful attention should be given to these beam modeling parameters. The mean MLC Gap and TGi complexity metrics were best suited to identifying clinical plans most sensitive to beam modeling errors; this could help provide focus for clinical QA in identifying unacceptable plans.

## 1 INTRODUCTION

The accuracy with which a treatment planning system (TPS) is commissioned determines how well, and how robustly, its dose calculations for simulated dose delivery represent the actual delivered dose distribution during radiotherapy treatments.

While every instance of atypical beam modeling does not necessitate a change in modeling parameters, there is evidence that parameter values different from community median values can be a cause for concern.^1^, ^2^ Previous studies have examined the impact multi‐leaf collimator (MLC) modeling errors can have on dose calculation accuracy and found clinically impactful dose errors.^3^, ^4^, ^5^, ^6^, ^7^, ^8^, ^9^, ^10^ However, these studies have been based on arbitrary parameter values and therefore may not reflect the true realities of clinical TPS modeling errors. The range of TPS modeling by the community was recently compiled and published by the Imaging and Radiation Oncology Core (IROC).^1^ A related study evaluated the dosimetric impact of atypical, but clinically used, TPS parameters (particularly the 2.5 and 97.5 percentile values) on IROC's IMRT head and neck (H&N) phantom.^11^ This study showed that several parameters, when substantially deviating from the median, resulted in dramatic dose deviations to the target. This is particularly concerning because TPS modeling errors have been identified in two‐thirds of all failing phantom results and the clinical use of such values was correlated with failing to correctly irradiate the IROC phantom.^11^, ^12^

There have been many studies conducted on how various beam modeling parameters can impact simulated dose delivery in phantoms or clinical cases using various arbitrary values.^3^, ^11^, ^13^, ^14^, ^15^ However, what remains unstudied, but also clearly warrants attention, is to better understand how the spread of TPS parameter values, and the use of atypical values, based on the actual clinical distribution of radiotherapy community data, affects the dosimetric accuracy of clinical patient plans. The magnitude of the impact that dosimetric or physical TPS beam modeling errors (based on current radiotherapy community practices) have on the accuracy of simulated dose delivery in clinical cases is currently unknown. A community data‐driven approach will yield results that can improve upon our current understanding and quantify the impact these modeling errors have on clinical practice.

This evaluation of plan sensitivity to modeling parameters may allow for a related evaluation of plan complexity metrics. Many complexity metrics have been proposed, and studies have examined the ability of complexity metrics to relate plan complexity and dose calculation accuracy.^16^, ^17^, ^18^, ^19^, ^20^ However, little has been done to determine which complexity metrics most comprehensively describe clinical plan sensitivity to known sources of dose calculation error, including TPS beam modeling parameters.

Therefore, this study sought to provide clinically relevant insight into the impact of beam modeling variability on the accuracy of simulated dose delivery, and to evaluate which complexity metrics best capture this relationship. By introducing differences in TPS parameters that are driven by radiotherapy community clinical values, we were able to determine which beam modeling errors translated into clinically relevant dose calculation errors in patient plans. The evaluation of complexity metrics provides novel and clinically guided insight into which complexity metrics best describe clinical plan sensitivity to TPS beam modeling parameters.

## 2 METHODS

### 2.1 Clinical plan selection

Twenty‐five standard fractionated IMRT and VMAT clinical cases, including five of each of prostate, non‐small cell lung, head and neck (oropharynx), mesothelioma, and brain plans, were retrospectively selected under institutional Internal Review Board protocol 2020‐0683. We identified plans in the RayPlanning clinical database (used for treatment planning) and then transferred them to the RayPhysics research system (beam commissioning module) to run all of our perturbations outside of the daily clinical workflow (both modules are within the RayStation TPS platform). Once transferred, we evaluated each plan to identify differences in the simulated dose delivery between RayPlanning and RayPhysics. The differences were less than 0.5% in the mean dose to clinical target volumes (CTVs), and preserved prescription dose coverage, average, minimum and maximum dose, and dose to 95% of the target volume. The plans were then recalculated (without re‐optimization), with fixed monitor units (MU), in RayPhysics version 10B with collapsed cone algorithm, on a Varian Millennium 120 MLC (21EX) Clinac machine at 6 MV. The beam model was built on a clinically commissioned TPS, using the radiotherapy community median TPS data (50‐percentile level for every parameter) for non‐dosimetric parameter values and linac specific reference data collected from IROC's site visit program for dosimetric values.^1^, ^12^, ^21^ This established a baseline for the simulated dose delivered to CTV and organs at risk (OAR) for each clinical plan.

### 2.2 TPS model parameter sensitivity study

The beam model built using the radiotherapy community median TPS data was used to create 40 additional beam models. Each subsequent beam model was identical to the 50‐percentile baseline version except for one parameter perturbation. The following 10 beam model parameters (the first seven are physical and the final three are dosimetric) were modified to match radiotherapy community data at the 2.5, 25, 75, and 97.5 percentile levels: Physical [multi‐leaf collimator offset (MLC offset), transmission (MLC transmission), gain (MLC gain; minor MLC offset tuning), curvature (MLC curvature; second order MLC offset correction), source size, leaf tip width (transmission along leaf through the rounded end), tongue and groove (interleaf leakage)], Dosimetric [percent depth dose (PDD), output factors, and off‐axis factors].^22^, ^23^, ^24^

Each clinical plan was recalculated on the 40 different beam models, generating a total of 1,000 perturbations, a sample of the parameters is shown in Table 1 (leaf‐tip width is included as it was a significant component in the complexity study).^21^ The difference in the mean planned dose to the CTV and parallel OAR, and the maximum dose (D_0.02_) to the serial OAR, were then evaluated with respect to the planned dose using the reference beam model (50‐percentile parameter values) to determine the clinical impact each parameter perturbation had on calculated dose, and therefore simulated dose delivery. Additional analysis was also done to determine how changes to the beam modeling parameters affect minimum and maximum dose, changes to the prescription coverage, and dose to 95% of the target structures (*D*_95_).

**TABLE 1:** Parameter values that demonstrated the greatest impact on simulated dose delivery (based on radiotherapy community data) and those used to evaluate the interplay between plan complexity and plan sensitivity to atypical beam modeling.

Non‐dosimetric parameters	2.5 percentile values	25th percentile values	50th percentile values	75th percentile values	97.5 percentile values
MLC offset (cm)	0.000	0.017	0.040	0.055	0.116
MLC transmission (%)	0.007	0.015	0.018	0.022	0.025
Leaf tip width (cm)	0.177	0.200	0.320	0.400	0.500

### 2.3 Complexity study

To determine the relationship between plan complexity and plan sensitivity to modeling errors, we focused on the parameters that had the greatest impact on dose calculation accuracy. We tabulated the number of times that each of the one thousand parameter perturbations introduced greater than ± 1% dose deviation in the mean CTV dose. The dose deviations greater than ± 1% were then grouped by parameter. The number of deviations for each parameter was compared to the grand total number of deviations greater than ± 1% across all 10 parameters. Only parameters that were responsible for at least 10% of the grand total number of plan perturbations (that demonstrated greater than ± 1% dose deviation in the mean CTV dose) were considered for the complexity study.

The following 18 complexity metrics were considered: modulation complexity score, modulation index total, plan irregularity, plan modulation, edge metric, leaf travel/arc length, mean tongue and groove index, MLC interdigitation, mean MLC speed, MLC speed modulation, mean dose rate, dose rate modulation, mean gantry speed, gantry speed modulation, mean MLC gap, first quartile of the distribution of the MLC gap sizes, MU, and number of arcs. These metrics were averaged across all beams in each plan and were then extracted using in‐house created PlanAnalyzer software.^25^ The metrics were obtained using software developed by a working group of the Catalan Society of Medical Physicists. The software was written in MATLAB (MathWorks, Inc.) and calculates complexity metrics using the data contained in the DICOM plan.^25^, ^26^

For each complexity metric, the correlation between the complexity score and the maximum CTV dose differences (across the 2.5, 25, 75, and 97.5 percentile levels) for each parameter was evaluated using linear regression. The complexity metrics that best fit the clinical data were selected based on the greatest average *R*‐squared value. Different numerical ranking systems, weighted *R*‐squared values, and root mean squared values were used to confirm the findings based on average *R*‐squared values.

## 3 RESULTS

### 3.1 TPS modeling parameter sensitivity study

**FIGURE 1:**
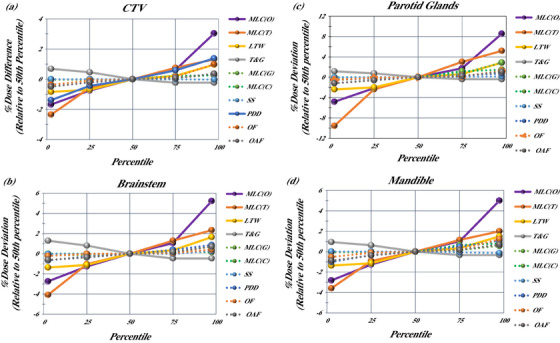
Dose perturbations relative to baseline 50th percentile values: (a) average mean dose to the CTV across all anatomical sites, (b) average maximum dose to the brainstem in brain plans, (c) average mean dose to the parotid glands across H&N plans, and (d) the average maximum dose to the mandible in H&N plans. LTW, leaf tip width; MLC(C), MLC curvature; MLC(G), MLC gain; MLC(O), multi‐leaf collimator offset; MLC(T), MLC transmission; OAF, off‐axis factor; OF, output factor; PDD, percent depth dose; SS, source size; T&G, tongue and groove.

The delivered dose simulated in the TPS showed changes in the average mean dose delivered to the CTV and average mean and maximum dose to selected OARs when TPS values were changed, as shown in Figure 1, for all parameters. For the CTV, this was averaged across all plans and anatomical sites and for OARs this was averaged across all anatomically relevant plans.

The MLC offset and MLC transmission parameters had the greatest impact on simulated dose delivery to CTVs and OARs. The MLC offset parameter, based on the community 2.5 percentile level, resulted in underdosing CTV volumes, on average, by 1.7% (max: 3.2%). The 97.5 percentile level resulted in overdosing CTV volumes, on average, by 3.0% (max 5.7%) and overdosing OAR structures, on average, by 6.2% (max 16.7%). The MLC transmission resulted in underdosing of the CTV volumes by 2.4% (max 5.0%) when the 2.5 percentile value was used, and the 97.5 percentile level resulted in overdosing CTV volumes by 1.3% (max 2.8%) and overdosing OAR structures by 3.6% (max 14.3%). Increased simulated dose delivery to OARs routinely elevated the dose above clinically acceptable limits based on these clinical scenarios. There were 200 plan perturbations for each of the five anatomical sites. Of the 200 H&N plan perturbations (all H&N plans were prescribed 70 Gy), 25 perturbations resulted in parotid glands receiving more than 26 Gy.^27^ Similarly, 41 of the 200 H&N cases resulted in the mandible receiving more than 70 Gy.^28^ In both instances, these changes were the result of parameter perturbations that represented 75 or 97.5 percentile community data values and in many cases overdosing exceeded 6%.

At a lower magnitude, perturbations to PDD also demonstrated an impact on simulated dose delivery. Changes to this parameter resulted in underdosing (when 2.5 percentile values were used) and overdosing (when 97.5 percentile values were used) CTV volumes, on average, by 1.4% (max 2.6%) and overdosing OAR by 1.1% (max 2.1%), where differences greater than 1.7% were found only in the prostate cases. The average dose deviations for the remaining seven parameters were all less than 1%.

**FIGURE 2:**
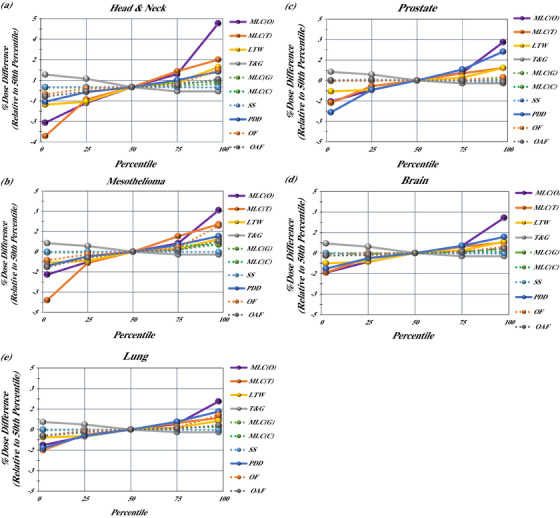
Changes in the average dose to the CTV, relative to the baseline 50th percentile average dose, by anatomical site: (a) H&N, (b) mesothelioma, (c) prostate, (d) brain, (e) lung.

To understand how these differences manifested across the five different anatomical sites, changes in the average mean simulated dose delivered to the CTV, relative to the baseline 50th percentile values, are shown in Figure 2 for each anatomical site. This figure highlights that MLC offset and MLC transmission are the two most impactful parameters for all anatomical sites.

While the trends are similar across all anatomical sites, there are differences in the magnitude of dose deviations as can be seen in Figure 2. CTV volumes for H&N and mesothelioma cases have the greatest sensitivity to MLC offset, followed by prostate, brain, and finally non‐small cell lung cases. The MLC transmission showed a similar trend, having the largest impact on H&N and mesothelioma cases, followed by prostate, non‐small cell lung, and finally brain cases.

**FIGURE 3:**
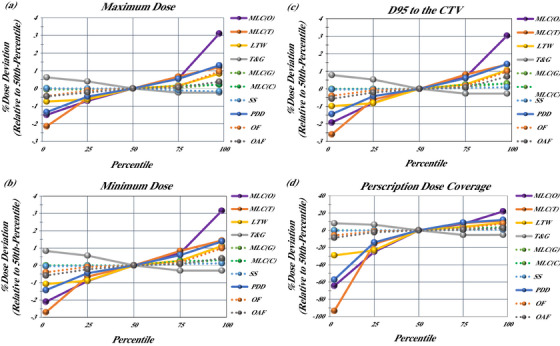
Changes in the average maximum, minimum, D95, and prescription dose coverage to the target structures, relative to the baseline 50th percentile dose, across all anatomical sites: (a) average minimum dose, (b) average maximum dose, (c) average D95, and (d) average prescription dose coverage.

We also examined the changes in the maximum, minimum, *D*_95_, and prescription dose coverage across the entire cohort (Figure 3) and found trends similar to observed changes in the mean CTV dose. These results reinforce that even when considering additional endpoints, that are commonly used in plan evaluation, the dose was most impacted by perturbations to the MLC Offset and MLC transmission parameters.

Changes to plan parameters most clearly affected the mean simulated dose delivered and induced systematic dose perturbations. Changes to parameter values had little effect on homogeneity, even with changes in off‐axis factors. There were a total of five instances, in a thousand cases, where plan perturbations resulted in ± 1% change in plan homogeneity. These instances occurred in mesothelioma or H&N plans for changes in the MLC offset or MLC transmission. This may be the result of most targets being of modest size and depth and centered on isocenter. Substantial dose deviations from perturbations to the off‐axis factor and PDD were only seen in the cases with larger tumors such as the head and neck and mesothelioma, or with deep tumors such as the prostate where the PDD perturbation was particularly important.

### 3.2 Complexity metrics

**FIGURE 4:**
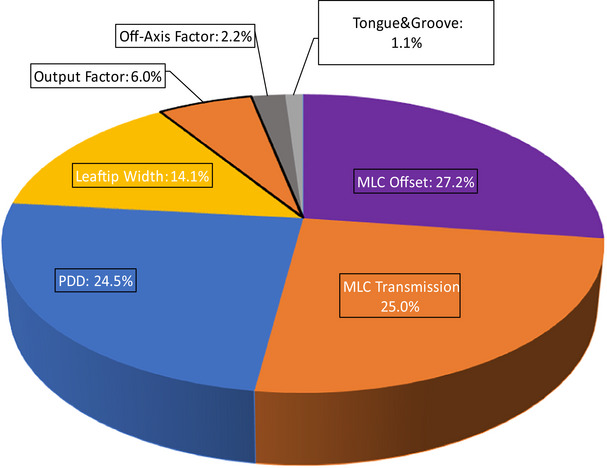
Relative contribution from each beam modeling parameter with a minimum impact of ± 1% to dose calculation accuracy.

The results from the TPS model parameter sensitivity study demonstrated an underlying difference in how each anatomical site was impacted by changes to modeling parameters. Thus, the relationship between plan complexity and plan sensitivity to modeling errors was evaluated. While the greatest impact on dose calculation accuracy (simulated dose delivery) was determined by the average dose deviations in both CTV and OAR structures (across the cohort) in the sensitivity study, we focused on the total number of instances that a parameter perturbation resulted in dose deviations of great than ± 1% for the complexity study. Of the 10 beam modeling parameters evaluated, perturbations to the seven parameters shown in Figure 4 caused dose deviations greater than 1% in CTV volumes; there were a total of 186 plans with such deviations out of the 1,000 plans.

The MLC offset, MLC transmission, PDD, and leaf‐tip width parameters comprised the vast majority of dose deviations greater than 1%. These were therefore included in the complexity metric evaluation, except for the PDD which was excluded as the impact of altering this parameter was found to rely on the geometry and anatomy of each case versus plan complexity.

**FIGURE 5:**
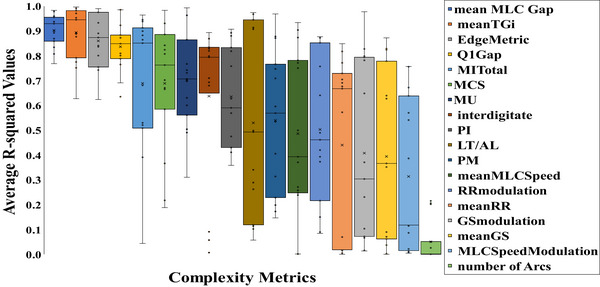
The legend corresponds to the complexity metrics in order from left to right (where the mean MLC Gap is shown to the far left and MLC Speed modulation is shown to the far right). The average *R*‐squared values represent the correlation between plan sensitivity to beam modeling errors and each of the 18 complexity metrics. The bottom line of each box represents the first quartile, the top line represents the third quartile, the line inside each box corresponds to the median value, “x” represents the mean value, and the vertical lines extend to the minimum and maximum values.

For each of the three relevant modeling parameters (MLC offset, transmission, and leaf‐tip width), the correlation between the maximum percent dose deviation in the CTV and the corresponding plan complexity metric score was evaluated using linear regression. The *R*‐squared values for correlation between each complexity metric score and the maximum percent dose deviation in CTV volumes were extracted, per anatomical site, for the MLC offset, MLC transmission, and leaf‐tip width beam modeling parameters (Figure 5). It is clear that different metrics have different predictive powers to identify dose perturbations associated with TPS modeling errors.

The mean MLC Gap (meanGap) and Tongue & Groove index (TGi) complexity metrics best fit the clinical data. The meanGap is the average leaf pair opening at each control point weighted by the corresponding fractions of MUs, which is related to the size of the MLC aperture. And the TGi is defined as the ratio of the difference between adjacent leaf positions and their MLC gap, averaged over all the leaves in the beam and all control points, which is related to the irregularity of the MLC aperture shape.^19^, ^29^ The complexity metrics remained constant despite the MLC parameter values used because complexity metrics depend on the plan characteristics, not on the MLC parameters used in the TPS configuration.

We observed a linear relationship between complexity values and the magnitude of dose deviation from the 50th percentile level. For example, plans with greater TGi values were more sensitive to parameter perturbations using 2.5 or 97.5 percentile values (e.g., resulting in a dose deviation of 4%), while plans with lower TGi values were less sensitive to parameter perturbations (e.g., resulting in a dose deviation of 2%). An approximate linear relationship between plan complexity values and changes in dose deviation from 50th percentile values was found, thus demonstrating that some complexity metrics can identify plans that are more sensitive to atypical beam modeling. Each additional ranking system identified the same complexity metrics as most appropriate for describing the relationship between plan complexity and plan sensitivity to beam modeling errors.

## 4 DISCUSSION

Our study quantified the impact of TPS beam modeling errors on patient dose calculation using TPS beam modeling parameter perturbations that match the current spread (for MLC and source terms) or error (for PDD and off‐axis and output factors) seen in radiotherapy community practices. And our results indicate that clinical delivery to our cohort would reveal errors in the delivered dose. Over or underdosing the CTV is problematic for clinical outcomes as is the overdosing of OAR. Variation in the MLC offset, MLC transmission, and PDD resulted in the greatest maximum dose difference across all anatomical sites, up to ± 5.7% and +16.7 to the CTV and OAR, respectively. Generally, the greatest dose impact occurred for the H&N plans, and the least occurred for the lung or brain plans. The meanGap and TGi were found to be the best complexity metrics at describing the impact of TPS beam modeling variations on clinical dose calculations across all sites. For these metrics, all anatomical sites showed similar trends between complexity and dose perturbation. In general, plans demonstrating greater complexity also exhibited greater dose deviations.

While not all atypical beam modeling values are necessarily incorrect, they are significantly associated with poorer performance and outright failure in IROC audits.^1^ It is expected that there will be some spread in community parameter selection to account for differences between machines, however, discrepancies of 1−2% (among matched or unmatched machines) are not large enough to account for the observed dose deviations with respect to 50‐percentile community values.^1^, ^21^, ^22^, ^30^, ^31^, ^32^, ^33^ A recent publication by Saez et al.^2^ found minimal differences between machine physical characteristic (less than 2%) compared to the differences in TPS modeling (resulting in differences of greater than 10% between calculated and measured doses). As such, we can expect that the differences in modeling will be much larger than the differences in the actual physical characteristics.^2^ Great care must be taken when using atypical values to ensure errors associated with suboptimal modeling are avoided, as they lead directly to errors in phantom audits and subsequently, incorrect dose in the patient. A comparison between selected and published 50‐percentile parameter values, followed by a dosimetry audit, can provide valuable insight as to the accuracy of beam model configuration.

The impact of dose differences due to parameter perturbations in beam modeling in this study are consistent with prior studies in both RayStation and Eclipse.^34^ Koger et al.^3^ found that in RayStation, parameters related to the modeling of the MLC had the greatest impact on dose calculation accuracy. Perturbations of ± 1 mm to the MLC offset parameter introduced dose deviations up to 10% in PTV structures and 15% in OARs for disease in H&N cases. Nithiyanantham et al.^35^ found that perturbations in MLC offset of 1 mm induced dose deviations up to 8.4% in the PTV and 10.8% in the OAR in anatomical sites similar to the current study. In our study, the 97.5 and 2.5 percentile values, corresponding to an offset of 1.1 mm, induced dose deviation of up to 5.7% and 16.7% in the CTV and OAR, respectively. As variations in TPS dose translate directly into changes in dose delivery, these results add the important and novel framework of evaluating the impact of TPS beam modeling errors on dose delivery based on the spread in TPS parameter selection currently seen by the radiotherapy community. These results are therefore especially relevant to the radiotherapy community at large.

This study directly generated numerical relationships between complexity scores and dose perturbations associated with atypical modeling parameters (Figure 5). Overall, plans with higher meanGap and lower TGi complexity scores were less sensitive to atypical beam modeling. It is therefore possible to provide an approximate guide to a complexity threshold that could maximize plan robustness by limiting dose errors even in the case of suboptimal beam modeling. In general, lung, brain, and prostate plans are less susceptible to beam modeling errors because they are less complex plans, while H&N and mesothelioma plans demonstrate greater sensitivity (Figure 2). Across anatomy, dose errors can be limited to less than approximately 2% if the meanGap is > 34.0 mm and the TGi is < 0.224 (as long as TPS parameters are not outside of the 97.5 percentile). Alternately, threshold values of > 20.0 mm and < .300 for the meanGap and TGi, respectively, would limit most dose errors to approximately 4%. If plan complexity scores are greater than these values, these plans will be particularly sensitive to suboptimal TPS modeling. Because different TPS and delivery platforms manifest complexity in different ways, these metrics are likely to be specific to the TPS/delivery platform evaluated: RayStation and Varian.^25^ A related limitation of this work is that complexity has been shown to manifest differently on different TPS/linac combinations.^22^ Therefore, while this work is descriptive of a common clinical system, it is unclear how other platforms may behave. For example, while some evaluations have been done using an Elekta platform, further work is needed before clinical decisions can be made concerning complexity metric selection.^21^, ^36^

A wide range of complexity metrics have been proposed over the past several years in an effort to best predict plans that have, or might have, errors.^16^, ^17^, ^18^, ^19^, ^20^ In this study, we reviewed 18 complexity metrics and found that the best metrics (for the Varian/RayStation combination) to predict clinical plan sensitivity to TPS parameter perturbations were the meanGap and TGi. In general, we found that aperture shape‐based complexity metrics best captured the correlation between plan sensitivity to TPS modeling errors and plan complexity when compared to metrics that were based on: MU, fluence, gantry speed, dose rate variation, or the leaf distance traveled.

Additionally, in this study, we have focused on the correlation between plan complexity and TPS errors using five different anatomical sites; we did not evaluate how other sources of error might manifest or expand our cohort to include additional treatment sites. Further, while we evaluated how complexity predicts dose calculation accuracy, this study did not address deliverability accuracy, which is an issue that warrants further consideration. Therefore, these results should not be interpreted to be a complete and comprehensive review of complexity. Nevertheless, this study is an important step towards a better understanding of how complexity metrics relate to an important and prevalent failure mode, and how they may be used in the clinic to limit dose calculation errors.^36^ Moreover, other error modes, such as a delivery error arising because of incorrect physical MLC leaf positioning, might reasonably follow the same trends with complexity metrics as incorrectly commissioning the MLC offset, although further study is required to confirm this theory.^37^

## 5 CONCLUSION

The parameters related to the modeling of the MLC offset and MLC transmission exhibited a substantial impact on the *D*_95_, prescription dose coverage, minimum, maximum and mean dose to CTV and relevant critical structures; modeling of the PDD, leaf tip width, and output factors were also important for dose calculation accuracy. It was found that these dose perturbations were related to plan complexity. The mean MLC Gap and Tongue & Groove index complexity metrics were best suited to identifying clinical plans that are more sensitive to beam modeling errors.

Use of atypical parameter modeling values requires careful attention by clinical physicists. Physicists should pay particular attention to MLC modeling parameters, as they can cause substantial dose deviations in clinical plans. Further, it is possible that aperture‐based complexity metric scores may be utilized to limit plan complexity, which in turn may reduce the overall impact of potential errors in the beam model. Ideally the correct approach would be for everyone to have a very robust model. However, given the observed challenges on that topic, a complexity threshold can serve as a safeguard against modeling errors negatively impacting accurate dose delivery. Thus, allowing for the time and effort necessary for an institution to implement a more robust beam model. For the Varian Clinac series, the mean MLC Gap complexity metric was best suited for identifying thresholds that have the potential to be used in clinical practice to reduce the sensitivity of treatment plans to beam modeling uncertainties, thus increasing the accuracy of radiation therapy treatment delivery.

## AUTHOR CONTRIBUTIONS

Fre'Etta Brooks and Stephen F. Kry conceived the presented work. Mallory C. Glenn and Fre'Etta Brooks created the beam models, Victor Hernandez, Jordi Saez created the plan analyzer. Hunter Mehrens and Julianne M. Pollard‐Larkin assisted Fre'Etta Brooks with data collection. Analysis and interpretations were primarily conducted by Fre'Etta Brooks, Stephen F. Kry, Victor Hernandez, Jordi Saez, Julianne M. Pollard‐Larkin, Rebecca M. Howell, Christine B. Peterson, Christopher L. Nelson, and Catharine H. Clark. The manuscript was drafted primarily by Fre'Etta Brooks and Stephen F. Kry; however, all authors provided feedback before the final draft submission.

## CONFLICT OF INTEREST STATEMENT

The authors have no conflicts of interest to report.

## References

[1] Glenn MC , Brooks F , Peterson CB , et al. Photon beam modeling variations predict errors in IMRT dosimetry audits. Radiother Oncol. 2022;166:8‐14. doi:10.1016/j.radonc.2021.10.021 34748857 PMC8863621

[2] Saez J , Bar‐Deroma R , Bogaert E , et al. Universal evaluation of MLC models in treatment planning systems based on a common set of dynamic tests. Radiother Oncol. 2023;186. doi:10.1016/j.radonc.2023.109775 37385376

[3] Koger B , Price R , Wang D , Toomeh D , Geneser S , Ford E . Impact of the MLC leaf‐tip model in a commercial TPS: dose calculation limitations and IROC‐H phantom failures. J Appl Clin Med Phys. 2020;21:82‐88. doi:10.1002/acm2.12819 PMC7021005 31961036

[4] McVicker D , Yin F‐F , Adamson JD . On the sensitivity of TG‐119 and IROC credentialing to TPS commissioning errors. J Appl Clin Med Phys. 2016;17:34‐48. doi:10.1120/jacmp.v17i1.5452 PMC5690193 26894330

[5] Kim J , Han JS , Hsia AT , Li S , Xu Z , Ryu S . Relationship between dosimetric leaf gap and dose calculation errors for high definition multi‐leaf collimators in radiotherapy. Phys Imag Rad Onc. 2018;5:31‐36. doi:10.1016/j.phro.2018.01.003 PMC7807868 33458366

[6] Tatsumi D , Hosono MN , Nakada R , et al. Direct impact analysis of multi‐leaf collimator leaf position errors on dose distributions in volumetric modulated arc therapy: a pass rate calculation between measured planar doses with and without the position errors. Phys Med Biol. 2011;56(20):N237‐246. doi:10.1088/0031-9155/56/20/N03 21965281

[7] Mu G , Ludlum E , Xia P . Impact of MLC leaf position errors on simple and complex IMRT plans for head and neck cancer. Phys Med Biol. 2008;53(1):77‐88. doi:10.1088/0031-9155/53/1/005 18182688

[8] Oliver M , Gagne I , Bush K , Zavgorodni S , Ansbacher W , Beckham W . Clinical significance of multi‐leaf collimator positional errors for volumetric modulated arc therapy. Radiother Oncol. 2010;97(3):554‐560. doi:10.1016/j.radonc.2010.06.013 20817291

[9] Nelms BE , Chan MF , Jarry G , et al. Evaluating IMRT and VMAT dose accuracy: practical examples of failure to detect systematic errors when applying a commonly used metric and action levels. Med Phys. 2013;40(11):111722. doi:10.1118/1.4826166 24320430 PMC8353583

[10] Kumaraswamy LK , Schmitt JD , Bailey DW , Xu ZZ , Podgorsak MB . Spatial variation of dosimetric leaf gap and its impact on dose delivery. Med Phys. 2014;41(11):111711. doi:10.1118/1.4897572 25370625

[11] Glenn MC , Peterson CB , Howell RM , Followill DS , Pollard‐Larkin JM , Kry SF . Sensitivity of IROC phantom performance to radiotherapy treatment planning system beam modeling parameters based on community‐driven data. Med Phys. 2020;47(10):5250‐5259. doi:10.1002/mp.14396 32677052 PMC7689833

[12] Kerns JR , Stingo F , Followill DS , Howell RM , Melancon A , Kry SF . Treatment planning system calculation errors are present in most imaging and radiation oncology core‐Houston phantom failures. Int J Radiat Oncol Biol Phys. 2017;98(5):1197‐1203. doi:10.1016/j.ijrobp.2017.03.049 28721904 PMC5567850

[13] Duchaine J , Wahl M , Markel D , Bouchard H . A probabilistic approach for determining Monte Carlo beam source parameters: iI. Impact of beam modeling uncertainties on dosimetric functions and treatment plans. Phys Med Biol. 2022;67(4):045006. doi:10.1088/1361-6560/ac4efb 35081514

[14] Paganini L , Reggiori G , Stravato A , et al. MLC parameters from static fields to VMAT plans: an evaluation in a RT‐dedicated MC environment (PRIMO). Radiat Oncol. 2019;14(216). doi:10.1186/s13014-019-1421-y PMC6889207 31791355

[15] Petersen N , Perrin D , Newhauser W , Zhang R . Impact of multileaf collimator configuration parameters on the dosimetric accuracy of 6‐MV intensity‐modulated radiation therapy treatment plans. J Med Phys. 2017;42(3):151‐155. doi:10.4103/jmp.JMP_88_16 28974861 PMC5618462

[16] Masi L , Doro R , Favuzza V , Cipressi S , Livi L . Impact of plan parameters on the dosimetric accuracy of volumetric modulated arc therapy. Med Phys. 2013;40(7):071718. doi:10.1118/1.4810969 23822422

[17] Dai J , Zhy Y . Minimizing the number of segments in a delivery sequence for intensity‐modulated radiation therapy with a multileaf collimator. Med Phys. 2001;28(10):2113‐2120. doi:10.1118/1.1406518 11695773

[18] McNiven AL , Sharpe MB , Purdie TG . A new metric for assessing IMRT modulation complexity and plan deliverability. Med Phys. 2010;37(2):505‐515. doi:10.1118/1.3276775 20229859

[19] Zygmanski P , Kung JH . Method of identifying dynamic multileaf collimator irradiation that is highly sensitive to a systematic MLC calibration error. Med Phys. 2001;28(11):505‐515. doi:10.1118/1.1408284 11764025

[20] Hernandez V , Hansen CR , Widesott L , et al. What is plan quality in radiotherapy? The importance of evaluating dose metrics, complexity, and robustness of treatment plans. Radiother Oncol. 2020;153:26‐33. doi:10.1016/j.radonc.2020.09.038 32987045

[21] Glenn MC , Peterson CB , Followill DS , Howell RM , Pollard‐Larkin JM , Kry SF . Reference dataset of users’ photon beam modeling parameters for the Eclipse, Pinnacle, and RayStation treatment planning systems. Med Phys. 2020;47(1):282‐288. doi:10.1002/mp.13892 31667870 PMC6980266

[22] Kerns JR , Followill DS , Lowenstein J , et al. Agreement between institutional measurements and treatment planning system calculations for basic dosimetric parameters as measured by the Imaging and Radiation Oncology Core‐Houston. Int J Radiat Oncol Biol Phys. 2016;95(5):1527‐1534. doi:10.1016/j.ijrobp.2016.03.035 27315667 PMC5113287

[23] Chen S , Yi BY , Yang X , Xu H , Prado KL , D'Souza WD . Optimizing the MLC model parameters for IMRT in the RayStation treatment planning system. J App Clin Med Phys. 2015;16:322‐332. doi:10.1120/jacmp.v16i5.5548 PMC5690186 26699315

[24] Saez J , Hernandez V , Goossens J , De Kerf G , Verellen D . A novel procedure for determining the optimal MLC configuration parameters in treatment planning systems based on measurements with a Farmer chamber. Phys Med Biol. 2020;65(15):5517‐5538. doi:10.1088/1361-6560/ab8cd5 32330917

[25] Hernandez V , Saez J , Pasler M , Jurado‐Bruggeman D , Jornet N . Comparison of complexity metrics for multi‐institutional evaluations of treatment plans in radiotherapy. Phys Imag Radiat Oncol. 2018;5:37‐43. doi:10.1016/j.phro.2018.02.002 PMC7807588 33458367

[26] Jurado‐Bruggeman D , Hernandez V , Saez J , et al. Multi‐centre audit of VMAT planning and pre‐treatment verification. Radiother Oncol. 2017;124(2):302‐310. doi:10.1016/j.radonc.2017.05.019 28687395

[27] Hey J , Setz J , Gerlach R , et al. Parotid gland‐recovery after radiotherapy in the head and neck region ‐ 36 months follow‐up of a prospective clinical study. Radiat Oncol. 2011;6:125. doi:10.1186/1748-717X-6-125 21951317 PMC3201902

[28] Wang X , Hu C , Eisbruch A . Organ‐sparing radiation therapy for head and neck cancer. Nat Rev Clin Oncol. 2011;8(11):639‐648. doi:10.1038/nrclinonc.2011.106 21788974

[29] Vieillevigne L , Khamphan C , Saez J , Hernandez V . On the need for tuning the dosimetric leaf gap for stereotactic treatment plans in the Eclipse treatment planning system. Radiat Oncol Phys. 2019;20(7):68‐77. doi:10.1002/acm2.12656 PMC6612699 31225938

[30] Glide‐Hurst C , Bellon M , Foster R , et al. Commissioning of the Varian TrueBeam linear accelerator: a multi‐institutional study. Med Phys. 2013;40(3):031719. doi:10.1118/1.4790563 23464314

[31] Isono M , Akino Y , Mizuno H , Tanaka Y , Masai N , Yamamoto T . Inter‐unit variability of multi‐leaf collimator parameters for IMRT and VMAT treatment planning: a multi‐institutional survey. J Radiat Res. 2020;61(2):307‐313. doi:10.1093/jrr/rrz082 31927580 PMC7246067

[32] Ghazal M , Södergren L , Westermark M , Söderström J , Pommer T . Dosimetric and mechanical equivalency of Varian TrueBeam linear accelerators. J Appl Clin Med Phys. 2020;21(12):43‐53. doi:10.1002/acm2.13058 PMC7769408 33070456

[33] Bhangle JR , Narayanan VK , Kumar NK , Vaitheeswaran R . Dosimetric analysis of beam‐matching procedure of two similar linear accelerators. J Med Phys. 2011;36(3):176‐180. doi:10.4103/0971-6203.83497 21897563 PMC3159224

[34] Vial P , Oliver L , Greer PB , Baldock C . An experimental investigation into the radiation field offset of a dynamic multileaf collimator. Phys Med Biol. 2006;51(21):5517‐5538. doi:10.1088/0031-9155/51/21/009 17047267

[35] Nithiyanantham K , Mani GK , Subramani V , Mueller L , Palaniappan KK , Kataria T . Analysis of direct clinical consequences of MLC positional errors in volumetric‐modulated arc therapy using 3D dosimetry system. J Appl Clin Med Phys. 2015;16(5):296‐305. doi:10.1120/jacmp.v16i5.5515 26699311 PMC5690184

[36] Chiavassa S , Bessieres I , Edouard M , Mathot M , Moignier A . Complexity metrics for IMRT and VMAT plans: a review of current literature and applications. Br J Radiol. 2019;92(1102):20190270. doi:10.1259/bjr.20190270 31295002 PMC6774599

[37] Lehmann J , Hussein M , Barry M , et al. SEAFARER—A new concept for validating radiotherapy patient specific QA for clinical trials and clinical practice. Radiother Oncol. 2022;171:121‐128. doi:10.1016/j.radonc.2022.04.019 35461949

